# Effects of different types of exercise intensity on improving health-related physical fitness in children and adolescents: a systematic review

**DOI:** 10.1038/s41598-024-64830-x

**Published:** 2024-06-21

**Authors:** Xianxian Zhou, Jiayu Li, Xiaoping Jiang

**Affiliations:** College of Physical Education and Health Sciences, Zhejiang Normal University, Jinhua, 321004 Zhejiang China

**Keywords:** Exercise intensity, Physical fitness, Children, Adolescents, Systematic review, Health services, Public health

## Abstract

A substantial body of empirical evidence reveals that physical activity is associated with a wide range of positive physical and mental health outcomes. However, an absence of comprehensive syntheses is observed concerning the varying effects of different exercise intensities on the improvement of physical health among children and adolescents. The aim of this review is to systematically investigate the effects of different exercise intensities on the physical fitness of children and adolescents, to analyses the optimal exercise intensities for improving physical fitness, and to provide a relevant theoretical basis for optimizing school physical education curricula. A systematic search strategy was used in this study in four online databases (PubMed, Scopus, EBSCO and Web of Science). Intervention studies that met the inclusion criteria underwent a thorough screening process, and their methodological quality was assessed utilizing the PEDro scale. The selected literature was systematically analyzed and evaluated through induction, summary, analysis, and evaluation. These findings indicate that high-intensity exercise training exerts significant positive effects on body composition, cardiopulmonary function and muscle fitness in children and adolescents. Therefore, we suggest that schools should focus on high-intensity sports in their physical education curriculum, which can further improve the student's PHYSICAL FITNESS.

## Introduction

Overweight and obesity in children and adolescents have become a global public health problem^1^. The prevalence of obesity in children and adolescents has been reported to have increased from 0.7% to 5.6%^2^. The persistence of overweight and obesity into adulthood has the potential to lead to chronic diseases, including type 2 diabetes, cardiometabolic disorders, and a range of psychosocial problems^3–6^, Numerous studies have shown that physical activity is one of the most important interventions to reduce physical health and psychological problems in adolescents^7–9^. WHO recommends that children and adolescents should engage in an average of 60 min of moderate to high-intensity physical activity (MVPA) per day to obtain health benefits^10^, however, more than 80%of adolescents fail to reach the minimum recommended amount of physical activity^11^. Given that adolescents have difficulty starting and following recommended guidelines for 30–60 min of moderate-intensity training per day^12,13^, there is a need to explore and develop engaging alternatives for youth to achieve the many health benefits of regular physical activity. Traditionally, moderate-intensity continuous training (MICT) has been the most common type of exercise recommended to improve body composition and cardiorespiratory fitness (CRF)^14,15^. However, in recent years, a growing body of laboratory evidence has shown that high-intensity exercise training is less time-consuming than MICT in improving body composition and other health indicators in obese children and adolescents^16–18^. Whether high-intensity or low-intensity exercise training is more beneficial to the PHYSICAL FITNESS of children and adolescents is still highly debated. Therefore, there is a need to further explore differences in the effectiveness of different exercise intensity interventions in improving PHYSICAL FITNESS in children and adolescents.

PHYSICAL FITNESS is a multidimensional state of being. PHYSICAL FITNESS is the body’s ability to function efficiently and effectively. It is a state of being that consists of at least FIVE HEALTH-RELATED and SIX SKILL-RELATED PHYSICAL FITNESS COMPONENTS, each of which contributes to total quality of life. The five components of health-related PHYSICAL FITNESS are BODY COMPOSITION, CARDIOVASCULAR FITNESS, FLEXIBILITY, MUSCULAR ENDURANCE, AND STRENGTH^19^. A recent narrative and meta-analysis of 20 studies evaluated the efficacy of HIIT for improving HEALTH-RELATED FITNESS (ie, cardiorespiratory fitness, muscular fitness, body composition and flexibility). The results indicated significant improvements in cardiorespiratory fitness and body composition through HIIT, with notable effects observed in these areas^13^. Previous meta-analyses have weakened the interpretation of findings due to small sample sizes. Furthermore, there is less research on exercise interventions to treat PHYSICAL FITNESS in children and adolescents than in adults, particularly in terms of exploring exercise-related variables (intensity and duration).

Therefore, this systematic review aims to systematically summarized the effects of different exercise intensities on health-related fitness in children and adolescents and to analyze which exercise intensity is more conducive to improving health-related fitness in children and adolescents.

## Methods

### Protocol

This review was performed according to Preferred Reporting Items for Systematic Reviews and Meta-analysis (PRISMA) guidelines^20^, and the Cochrane Handbook for systematic review^21^. The PRISMA checklist is presented in Additional File 1.

### Search strategy

A comprehensive search was done systematically through PubMed, Scopus, EBSCO, and Web of Science up to the 5 of June 2024. Searching terms were based on adapted PICO questions to search through the aforementioned databases to access all the important articles. Free text words and medical subject heading (MeSH) terms were used. (1) children OR childhood OR pre*schooler OR schoolchildren OR preadolescent OR adolescent OR adolescence OR youth;(2) physical*activity OR physical*education OR exercise OR fitness OR sport;(3) strength OR flexibility OR motor OR endurance OR agility OR body composition OR anthropometry OR body mass index OR waist circumference OR overall adiposity OR central adiposity OR overweight OR obesity OR risk factors OR risk score cardiovascular disease OR metabolic syndrome OR blood glucose OR glucose tolerance OR insulin resistance OR insulin sensitivity OR blood lipids OR dyslipidemia OR diabetes OR blood pressure OR hypertension OR inflammatory markers OR bone mineral OR bone mineral content;(4) random OR random*controlled trial OR controlled trial OR trial. (The search strategy used for each database is provided in the supplementary material (table S2). At the same time, the reference lists of included articles and relevant reviews were retrospectively included to supplement the missing literature in the computer search. The systematic search process was conducted by XXZ and JYL. Any disagreement of an included/excluded study was resolved by the author PXJ.

### Eligibility criteria of the selected studies

The inclusion criteria for articles were determined using the PICOS (Participants/Interventions/Comparisons/Outcomes/Study Design) principles, as follows. Participants (P): Children and adolescents (individuals in the 10–19 year age group^22^, including samples of overweight/obese children, but excluding samples of children with medical conditions); Interventions (I):interventions in the form of exercise, High-intensity aerobic exercise, Low-intensity aerobic training (LIT), Endurance training (ET), High-intensity interval exercise (HIIE), Moderate-intensity exercise (MIE),HIIT, moderate-intensity continuous (MICT); Comparisons (C): control group performed low to moderate intensity physical activity or no artificially designed physical exercise; Outcomes (O): assessment of at least one of the following indicators (i.e., body composition, cardiorespiratory fitness, muscular fitness, strength, flexibility, motor, endurance, agility, body composition, anthropometry, body mass index, waist circumference, overall adiposity , central adiposity , overweight , obesity ,risk factors , risk score cardiovascular disease, metabolic syndrome, blood glucose, glucose tolerance, insulin resistance, insulin sensitivity, blood lipids, dyslipidemia, diabetes, blood pressure, hypertension, inflammatory markers, bone mineral, bone mineral content); Study Design (S): controlled trial.

Exclusion criteria: (1) studies not related to the topic (non-physical activity, physical activity); (2) non-intervention studies (observational studies, systematic reviews) and studies that did not provide sufficient comparisons to compare; (3) Exclude other age groups other than 3–19 years old. The title, abstract and full text were independently assessed by two authors for eligibility. Finally, randomized controlled trials were limited to articles published in English.

### Data extraction

Data extraction from the included studies was independently performed by two authors (XXZ and JYL). For each study, data were extracted for the characteristics of the study population. These include (1) first author’s surname; (2) year of publication; (3) purpose; (4) results; (5) the characteristics, sample size and age of the participants; (6) sampling type; (7) type of research; (8) Characteristics of physical exercise (type, frequency and duration). Any disagreement in data extraction was resolved by the third author PXJ (Table 1).

**Table 1:** Study characteristics.

References	Results	Sample characteristics	Research design	Intervention measure
Farah et al. ^24^	HIT has additional benefits for abdominal obesity and cardiovascular health compared to LIT	43 obese adolescents (M = 15.4 ± 0.4 years)	Randomly assigned	6 months of HIT and LIT training, three times a week
Hay et al. ^25^	High-intensity ET improves Cardiopulmonary function in obese adolescents, however, the effect of exercise intensity on insulin sensitivity and triglycerides is unclear due to a lack of adherence	106 overweight and obese adolescents (M = 15.2 ± 0 years)	Randomly assigned	6 months of high- or moderate-intensity ET, 2 times a week, 40 min
Bond et al. ^26^	In the adolescent group, HIIE performed provided better vascular benefits than MIE	20 adolescents (M = 14.3 ± 0.3 years)	Randomly assigned	Cycling interventions of varying intensity
Paravidino et al. ^27^	Aerobic exercise can change the level of spontaneous physical activity in overweight adolescents, and overweight adolescents should be encouraged to engage in moderate- to high-intensity physical activity to promote negative energy balance and promote weight loss	24 overweight adolescents (M = 12.6 ± 0.95 years)	Randomly assigned	Each exercise lasts 60 min, with walking and running at different intensities crossed
Tadiotto et al. ^28^	HIIT reduces BMI-z, waist-to-height ratio, and improves physical fitness	52 adolescents (11–16 years)	Non-randomly allocation	12 weeks of HIIT and moderate-intensity interval training
Larsen et al.(2018)^29^	A well-organized high-intensity physical education program can make a positive contribution to the healthy musculoskeletal development of young children	295 adolescents (M = 10.0 ± 0.3 years)	Randomly assigned	3 × 40 min of SSG or CST per week
Ramirez-Velez etal. ^30^	Based on the LIPE and groups, several levels of circulating inflammation can be significantly altered	95 adolescents (M = 13.5 ± 1.6 years)	Randomly assigned	School-based exercise programs. 3 times a week for 6 months
Cao et al. ^31^	HIIT was highly effective in improving cardiopulmonary function fitness compared to MICT and had a similar effect in improving body composition in obese boys. In addition, HIIT also effectively reduces visceral adipose tissue, which is more time-effective than MICT	45 obese adolescents (M = 11.2 ± 0.7 years)	Randomly assigned	12 weeks of school running training at different exercise intensities
Dias et al. ^32^	Compared to MICT, HIIT is very effective in improving cardiopulmonary function	99 obese children (7–16 years)	Randomly assigned	Training 3 times a week for 12 weeks of HIIT and MIT training
Faigenbaum et al. ^33^	Different training regimens can improve muscle strength and muscular endurance in children, and high-repetition-moderate-load training may be more beneficial than low-repetition-heavy-load training	43 children (M = 5.2 ± 11.8 years)	Randomly assigned	8 weeks of non-consecutive daily training twice a week resistance training program
Benson et al. ^34^	In normal-weight and overweight children, PRT at 8 weeks significantly improved central and generalized obesity associated with muscle strength	78 children (M = 12.2 ± 1.3 years)	Randomly assigned	PRT training twice a week for 8 weeks
Taber et al. ^35^	Adolescent girls' participation in vigorous exercise rather than moderate exercise was positively associated with cardiopulmonary function fitness	1019 adolescents (M = 13.99 ± 0.53 years)	Non-randomly allocation	Moderate and vigorous exercise for 12 months
Davis et al. ^36^	After 13 weeks of intervention, aerobic training of 20 or 40 min/day improved physical performance in sedentary overweight or obese children	209 children (M = 9.4 ± 0.1 years)	Randomly assigned	20 min or 40 min of aerobic training daily for 13 weeks
Burns et al. ^37^	Acute sprinting interval exercise can lead to short-term increased oxygen intake and decreased blood pressure in young people	10 adolescents (M = 17.2 ± 0.7 years)	Randomly assigned	High-intensity sprint intervals on a bicycle ergometer
Leppanen et al. ^38^	High-intensity exercise is not only associated with higher physical fitness and massless fat index but also effectively improves children's body composition	307 children (4 years)	Randomly assigned	Six months of exercise interventions of varying intensity
Leppanen et al. ^39^	Promoting high-intensity physical activity in preschool-age has long-term beneficial effects on children's body composition and fitness, especially muscle strength	315 children (4 years)	Non-randomly allocationr	Six months of exercise interventions of varying intensity
Gomes et al. ^40^	Multidisciplinary interventions are effective in regulating body composition and obesity in adolescents. composition	42 obese adolescents (13–17 years)	Non-randomly allocationr	A multidisciplinary intervention lasting 12 weeks
Buchan et al. ^41^	HIT interventions can be used in adolescent school settings as a means of improving physical fitness	89 adolescents (M = 16.7 ± 0.6 years)	Randomly assigned	Exercise three times a week for 7 weeks
Grasten et al. ^42^	Physical activity of moderate to vigorous intensity is positively associated with cardiopulmonary function fitness	446 children (M = 11.26 ± 0.32 years)	Non-randomly allocation	A three-year intervention study
Costigan et al. ^43^	AEP and RAP had moderate intervention effects on participants' waist circumference and BMI. The resistance and cardiopulmonary function of the aerobic exercise program group had a significant small intervention	65 adolescents (M = 15.8 ± 0.6 years)	Randomly assigned	The eight-week intervention included an aerobic exercise program, resistance, and aerobic exercise three times a week
Saidi et al. ^44^	Acute intensive training increases sleep duration and sleep quality in young olive athletes without interfering with the next day's performance or dietary intake	17 adolescents (M = 15.7 ± 1.1 years)	Non-randomly allocation	Two 36-h lab sessions for high-intensity rugby training
Saidi et al. ^45^	Acute exercise effectively increases sleep duration and sleep quality in obese adolescent girls, thereby reducing subsequent high-energy food consumption	16 adolescents (M = 13.7 ± 1.1 years)	Randomly assigned	A 12-week athletic training program that includes 3 h of exercise per week
Gerber et al. ^46^	Higher moderate to heavy physical activity is associated with better cardiorespiratory fitness	2166 children (M = 8.0 ± 1.6 years)	Randomly assigned	physical activity
Winn et al. ^47^	Regardless of asthma status, HIIT is an effective tool for improving adolescent aerobic fitness and maintaining BMI	221 adolescents (M = 13.0 ± 1.1 years)	Randomly assigned	HIIT courses three times a week for 6 months
Paulino et al. ^48^	Introducing HIIT in the school setting has a high potential for improving physical fitness and has a moderate effect on improving body composition in adolescents	300 adolescents (15–17 years)	Randomly assigned	16-week, twice-weekly 90-min HIIT classes
Videira-Silva et al. ^49^	Although carotid darter-intimal thickness is impaired in overweight adolescents, improvements in overall lipid mass, moderate intensity, and cardiorespiratory function are associated with improvements in carotid darter-intimal thickness	105 adolescents (M = 14.8 ± 1.8 years)	Non-randomly allocation	6 months of cycling training, moderate intensity training
Juric et al. ^50^	The 12-week HIIT intervention was effective in adolescent cardiorespiratory function	87 adolescents (10–15 years)	Randomly assigned	12 weeks of 10-min HIIT sessions twice a week
Farpour-Lambert et al. ^51^	Regular physical activity can reduce blood pressure, and atherosclerosis, and delay arterial remodelling in obese children before puberty	44 obese children (M = 8.9 ± 1.5 years)	Randomly assigned	A three-month physical activity program
Ketelhut, Sascha et al. ^52^	School-based HIIT can induce improvements in cardiovascular parameters	40 students (M = 11 ± 1 years)	Randomly assigned	12 weeks of 20-min HIIT sessions twice a week
Migueles et al. ^53^	An aerobic plus resistance exercise program improved cardiometabolic health in children with overweight or obese but had no effect on mental health	96 students (M = 10 ± 1.1 years)	Randomly assigned	20 weeks of 90-min resistance exercise three a week

HIT, High-intensity aerobic exercise; LIT, Low-intensity aerobic training; BP, Blood pressure; HR, heart rate; HRV, Heart rate variability; ET, Endurance training; HFM, High-fat meals ; HIIE, High-intensity interval exercise; MIE, Moderate-intensity exercise; HIIT, high-intensity interval training; MICT, moderate-intensity continuous; WC, waistline; FM, Fat mass; FFM, Fat-free amount; BMI-z, BMI z-score; SSG, Small team ball game; CST, Circuit strength training; HIPE, High-intensity physical education classes LIPE, Low to moderate physical education PLUS, combination; P RT, High-intensity progressive resistance training BMI, Body mass index; AEP, Aerobic exercise program; RAP, Resistance and aerobic exercise programs; CI, Côte d Ivoire; ZA, South Africa; TZ, Tanzania; PA, Physical activity; CRF, Cardiopulmonary function. The measurement tools are detailed in Supplementary Table S3.

### Quality assessment

Papers that met the inclusion criteria were independently assessed by two authors (XXZ and JYL). This review assessed the included literature using the Physiotherapy Evidence Database (PEDro) scale, a credit rating scale developed by the Australian Centre for Evidence-Based Practice. The PEDro scale is a valid measure of the methodological quality of clinical trial^23^. The scale consisted of randomized grouping (2 items), blinding (3 item), data reporting (3 item), data analysis (1 item), and follow-up (1 item), with a total of 10 criteria. Each item was recorded as 1 point when it appeared in the article and 0 points when it was not reflected, for a total score of 0 to 10 points. To avoid subjective opinions, two reviewers assessed the opinions, and the third judged the differences. It classifies papers into three levels: high quality above 8, medium quality 4–7, and low quality below 4 points. Disagreements were solved by a third party (PXJ) (Table 2).

**Table 2:** Results of study quality evaluation of included studies.

Reference	Eligibility criteria	Random allocation	Concealed allocation	Groups similar at baseline	Participants blinded	Provider blinded	Evaluator blinded	Follow Up	Intention to-treat analysis	Between group Comparison	Pedro score
Farah et al. ^24^	1	1	0	1	0	0	0	1	1	1	Medium
Hay et al. ^25^	1	1	1	1	0	0	1	1	1	1	High
Bond et al. ^26^	1	1	0	1	0	0	0	1	1	1	Medium
Paravidino et al. ^27^	1	1	0	1	1	0	0	1	1	1	Medium
Tadiotto et al. ^28^	1	0	0	1	0	0	0	1	1	1	Medium
Larsen et al. ^29^	1	1	1	1	0	0	0	1	1	1	Medium
Ramirez-Velez etal. ^30^	1	1	1	1	1	1	1	1	1	1	High
Cao et al. ^31^	1	1	1	1	0	0	0	1	1	1	Medium
Dias et al. ^32^	1	1	0	1	0	0	0	1	1	1	Medium
Faigenbaum et al. ^33^	1	1	1	1	0	0	0	1	1	1	Medium
Benson et al. ^34^	1	0	0	1	0	0	0	1	1	1	Medium
Taber et al. ^35^	1	1	1	1	1	0	0	1	1	1	Medium
Davis et al. ^36^	1	1	0	1	0	0	0	1	1	1	Medium
Burns et al. ^37^	1	1	0	1	0	0	0	1	0	1	Medium
Leppanen et al. ^38^	1	0	0	1	0	0	0	1	0	1	Medium
Leppanen et al. ^39^	1	0	0	1	0	0	0	0	1	1	Medium
Gomes et al. ^40^	1	1	0	1	0	0	0	1	1	1	Medium
Buchan et al. ^41^	1	0	0	1	0	0	0	0	0	1	Low
Grasten et al. ^42^	1	1	0	1	0	1	0	1	1	1	Medium
Costigan et al. ^43^	1	0	0	1	0	0	0	1	1	1	Medium
Saidi et al. ^44^	1	1	0	1	0	0	0	1	1	1	Medium
Saidi et al. ^45^	1	1	0	1	0	0	0	1	1	1	Medium
Gerber et al. ^46^	1	1	0	1	0	0	0	1	1	1	Medium
Winn et al. ^47^	1	1	1	1	0	0	0	1	1	1	Medium
Paulino et al. ^48^	1	1	1	1	0	0	0	1	1	1	Medium
Videira-Silva et al. ^49^	1	1	1	1	0	0	0	1	1	1	Medium
Juric et al. ^50^	1	0	0	1	0	0	0	1	1	1	Medium
Farpour-Lambert et al. ^51^	1	1	0	1	0	0	0	1	1	1	Medium
Ketelhut, Sascha et al. ^52^	1	1	0	1	0	0	0	1	1	1	Medium
Migueles et al. ^53^	1	1	1	0	1	0	0	1	1	1	Medium

### Data synthesis and analysis

Due to the heterogeneity of the studies, no meta-analysis was performed. Instead, intervention characteristics for each study were summarized and analyzed and then recorded in a standardized form created by the authors. The effectiveness of the intervention was calculated using the formula: number of effective trials (post-intervention scores significantly higher than pre-intervention or control scores)/total number of trials. Data analysis was performed by the first author XXZ and then validated by the second author JYL.

## Results

### Literature screening process and results

**Figure 1:**
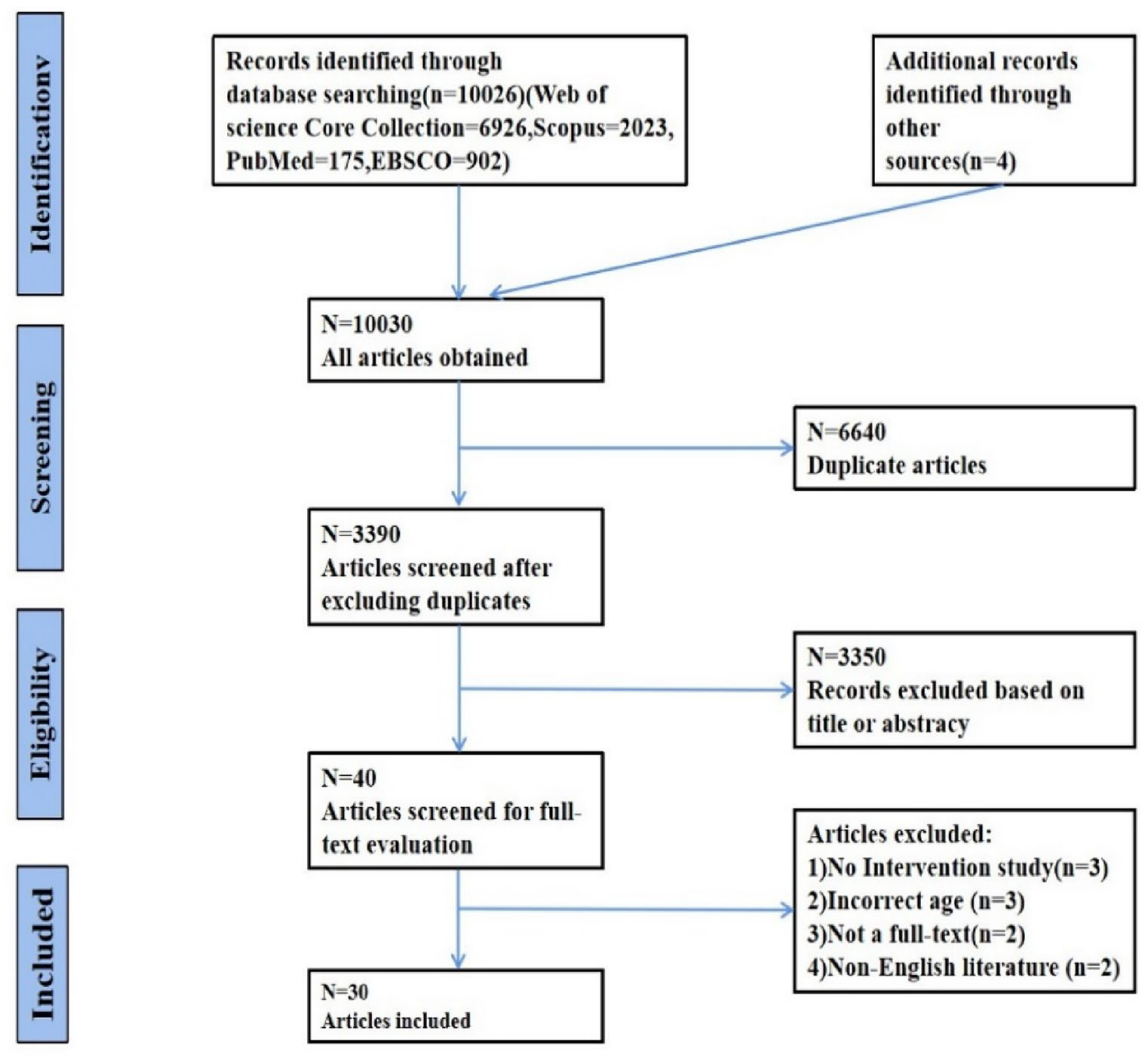
Flow chart of literature retrieval.

A preliminary search of the database yielded 10,030 relevant studies. We first imported the documents into the document management software Endnote, and after removing duplicate documents and screening titles and abstracts, we excluded 9990 articles. Of the remaining 40 articles, 30 articles were obtained after screening and checking the full text, and the irrelevant articles were eliminated. The reasons for the exclusion based on the full text were: (1) no intervention studies (3 articles); (2) The age does not meet (3 articles); (3) non-full text (2 articles); (4) non-English articles (2 articles). The PRISMA flowchart is shown in Fig. 1.

The systematic search of relevant literature published as of 5 June 2024 found 30 relevant articles, the earliest of which was published in 1999. The study included 30 related papers from the United States, Canada, Brazil, Denmark, Spain, China, Australia, the United Kingdom, Singapore, France, Portugal, Colombia, and Switzerland.

Study characteristics were summarized in Table 1, and the final analysis included 6494 children and adolescents with participants ranging in age from 5 to 18 years, with most studies including healthy children and adolescents, but nine studies including overweight or obese children. Study sizes ranged from 10 to 2166. Physical activity interventions mainly included HIIT (8/30; 27%), aerobic training (5/30; 17%), resistance training (2/30; 7%), physical education (1/30; 3%), endurance training (1/30; 3%), acute exercise (1/30; 3%) and other interventions. Outcome measures: body mass index, waist circumference, body fat, cardiorespiratory fitness, and muscle fitness (muscular endurance, muscle strength and muscular flexibility). Quality scores for 30 studies are shown in Table 2. The studies ranged in their scores from 3 to 10. Only two studies achieved high-quality scores (≥ 8) (Table 2). One study scored below 4. Blinding techniques ranged from 0 to 3 in this study, with only one study scoring 3 and four scoring 1; Fifteen studies scored 0.

### Effects of different exercise intensity on BODY COMPOSITION in children and adolescents

A total of 11 studies in this study assessed the effects of different exercise intensities on body composition (weight, BMI, body fat, waist circumference, fat-free mass, and other relevant indicators), of which 9 showed that high-intensity exercise interventions had a positive effect on overweight or obese children and adolescents, but 2 had no positive effect.

#### Weight, BMI and body fat

A total of 8 of the 11 studies assessed changes in body weight, BMI, adiposity, or percentage of body fat measured. Six of the eight studies, reported positive effects of high-intensity exercise interventions on body weight, BMI, or body fat in overweight and obese child adolescents. However, 2 studies showed moderate or no positive effects of high-intensity exercise interventions on BMI, and body fat in overweight or obese children and adolescents.

Tadiotto et al. conducted a 12-week HIIT and MIIT intervention study and found significant reductions in (body mass index) BMI-z, (waist-to-height ratio) WHtR, and LDL-c in HIIT^28^. Benson et al. compared the effects of high-intensity progressive resistance training (PRT) on body composition in obese children and showed that an 8-week PRT intervention resulted in significant improvements in adiposity, percentage body fat, and body mass index^34^. Recent findings have shown that after 12 weeks of HIIT and MICT interventions, there was a significant reduction in BMI and body fat mass in the HIIT group compared to the control group, as well as a significant reduction in visceral adipose tissue (− 53 g vs. − 17 g, *p* < 0.01), LDL cholesterol was reduced only in the HIIT group, whereas in MICT only the body fat percentage was significantly reduced (− 17.2%, *p* < 0.05)^31^. In addition, Winn et al. compared the effects of HIIT on adolescents over a 6-month period and showed that after a 6-month school HIIT intervention, BMI was maintained in the HIIT group and significantly increased in the control group, and that HIIT was an effective tool for maintaining BMI^47^.

In a study assessing the effect of different exercise intensities on energy expenditure for spontaneous physical activity in adolescents, Paravidino et al. found that the mean energy expenditure was 82, 286 and 343 kcal in the control, moderate and vigorous exercise groups, respectively (*p* < 0.001), and the results suggest that high intensities are more conducive to an increase in energy expenditure, and thus to weight loss^27^. Saidi et al. studied the effect of vigorous exercise on subsequent dietary intake in obese adolescent girls and showed a significant reduction in adiposity in the exercise group compared to the control group (*p* < 0.02)^45^.

In the present study, 2 studies reported no significant effects of different intensities of exercise on body composition in overweight or obese children and adolescents. Gomes et al. compared the effects of different aerobic training intensities over a period of 12 on the body composition of obese adolescents, and showed a decrease in body weight, BMI, and body fat in both the intervention and control groups after a 12-week intervention (*p* < 0.001), but these results could not be attributed solely to aerobic training intensity due to the multidisciplinary intervention^40^. In another study, Costigan et al. conducted an 8-week study of aerobic training (AEP) and resistance and aerobic programming (RAP) with 68 secondary school students, and the results showed a moderate effect of the BMI intervention for participants in the AEP and RAP groups. It may be related to the small sample size^43^.

#### Waist circumference

Three randomized controlled trials assessed changes in waist circumference and all found beneficial effects. (Insert literature), a study conducted by Farah et al., showed that after 6 months of high-intensity aerobic training (HIT) and low-intensity aerobic training (LIT), significant beneficial changes in waist circumference were found only in the HIT group^24^. Benson et al. investigated the effects of 8 weeks of high-intensity progressive resistance training (PRT) on body composition in obese children and compared the effects between the experimental and control groups, showing that significant changes in waist circumference were obtained in the intervention group after 8 weeks of PRT training^34^. Costigan et al. conducted an 8-week study of aerobic training (AEP), resistance and aerobic programming (RAP) with 68 secondary school students and showed that participants in the AEP and RAP groups had significant changes in waist circumference (*p* = 0.024)^43^.

#### Fat-free mass

Only 1 study evaluated the effect of different exercise intensities on fat-free mass. Leppanen et al. investigated the effect of physical activity intensity and sedentary behaviours (ST) on body composition in 4 years old children. The results showed that the higher the intensity of moderate-to-vigorous exercise, the lower the percentage of fat (%FM, *p* = 0.015), the VPA (high intensity) and MVPA (moderate-to-vigorous exercise intensity) the higher the fat-free mass index (FFMI, *p* = 0.002 and *p* = 0.011) Time spent on VPA was associated with higher FFMI^38^.

### Effects of different exercise intensities on CARDIOPULMONARY FUNCTION (CRF) in children and adolescents

A total of 16 studies investigated the effects of different exercise intensity interventions on cardiorespiratory fitness, and positive effects were found in all studies. In general, cardiorespiratory fitness improved with high-intensity exercise interventions. The included studies assessed vascularity, heart rate, lipids, insulin sensitivity, inflammatory markers, diabetes, and other relevant indicators.

#### Blood vessels

A total of 4 out of 16 studies investigated the effects of exercise intensity interventions on blood vessels in children and adolescents. Four studies demonstrated that high-intensity training interventions had a positive effect on blood vessels.

Bond et al. investigated the effect of exercise intensity on protecting the vascular system from high-fat diets in adolescents study by intervening with high-intensity interval exercise (HIIE) and moderate-intensity exercise (MIE) in 20 adolescents, and showed that exercise intensity plays an important role in protecting the vascular system from the deleterious effects of HFM, and that in the adolescent population, performing HIIE may be more effective than MIE in Provides better vascular benefits^26^. In a study examining the effects of sprint interval exercise on post-exercise metabolism and blood pressure in adolescents, it was shown that acute sprint interval exercise leads to an increase in short-term oxygen uptake and a decrease in blood pressure in adolescents^37^. Farpour-Lambert et al. investigated the effect of physical activity on systemic blood pressure in adolescent obese children, and after a 3-month intervention, significant changes in systolic and diastolic blood pressure were obtained in the intervention group compared to the control group^51^. Buchan et al. investigated whether a high-intensity training (HIT) intervention could improve the CVD risk profile of adolescents in a time-effective manner, and after a 7-week HIT intervention, a significant reduction in systolic blood pressure was obtained in the intervention group compared to the control group^41^.

#### Insulin sensitivity

Of the 16 studies, only 2 randomized controlled trials assessed the effect of exercise intensity on insulin sensitivity. Only one study showed that a high-intensity exercise intervention could have a positive effect on insulin sensitivity. In the first randomized controlled trial, the Davis study found that after the intervention, the high-dose aerobic training group had a greater reduction in insulin (AUC), which could be effective in reducing metabolic risk^36^. However, in another randomized trial of 106 overweight and obese adolescents who underwent high-intensity endurance training (ET) and moderate-intensity (ET) for 6 months, the results showed that ET significantly improved cardiorespiratory fitness in obese adolescents, but the effect of exercise intensity on insulin sensitivity and triglycerides remained unclear due to lack of compliance^25^.

#### Inflammation

A total of 3 out of 16 studies assessed the effect of exercise intensity interventions on inflammation, with only 2 showing a positive effect of high-intensity exercise interventions on the prevention of inflammation. The results of the study by Ramirez-Velez et al. suggest the utility of high-intensity aerobic and resistance training as a means of modulating the levels of certain pro-inflammatory interleukins in adolescent subjects, thereby playing an important role in the prevention of diseases associated with low-grade inflammation, such as cardiovascular disease and type 2 diabetes^30^. A study by Tadiotto et al. found that C-reactive protein (CRP) was significantly reduced in the HIIT group, promoting beneficial changes in obesity and inflammatory processes^28^. However, in a study conducted by Buchan et al. with 89 adolescent students to assess whether the HIIT intervention could improve the cardiovascular disease risk profile of secondary school students in a time-effective manner, after a 7-week intervention, the results showed no significant differences between groups for any of the nine biochemical risk markers for cardiovascular disease, but significantly improved cardiorespiratory fitness^41^.

#### Heart Rate

Two of the 16 studies showed that high-intensity exercise interventions had a positive effect on heart rate. In one study examining the effect of exercise intensity on blood pressure and heart rate in obese adolescents, after a 6-month period of HIT and LIT, beneficial changes in HR and HRV occurred only in the HIT group^24^. In a randomized controlled trial, Ketelhut et al. assessed the effect of implementing school-specific HIIT in a physical education curriculum on various hemodynamics parameters and heart rate variability, and after a 12-week intervention, the results showed that significant changes in heart rate were obtained in the intervention group (*p* = 0.010)^52^.

In addition, five other studies have all demonstrated the beneficial effects of high-intensity exercise interventions on cardiorespiratory fitness. Grasten et al. examined the effects of moderate-to-vigorous physical activity and ST with cardiorespiratory fitness in schoolchildren from 2017 to 2020, assessing accelerometer based MVPA by using waist-worn activity monitors and CRFs at four measurement points using the 20-m shuttle run test and ST, which showed a positive correlation between MVPA and CRF, and a negative correlation between ST and CRF^42^. Taber et al. conducted a moderate and vigorous exercise intervention with 1,029 eighth-grade girls and measured cardiorespiratory fitness using the Modified Physical Exercise Capacity Test (MPCT), which showed that vigorous exercise was positively associated with cardiorespiratory fitness^35^. Dias et al. showed that after 12 weeks of HIIT and MICT interventions, the HIIT group had a significant increase in relative peak VO2 compared to MICT, which was very effective in improving cardiorespiratory fitness^32^. Both studies by Gerber et al. and Leppanen et al. showed that higher levels of MVPA were associated with higher CRF scores^39^.

### Effects of different exercise intensities on FLEXIBILITY in children and adolescents

Only two studies assessed changes in flexibility and no effects were found. The first study, conducted by Buchan et al., showed that after a 7-week period of high-intensity interval exercise, the intervention group showed an increase in vertical performance, and 10-m sprint speed (*p* <  = 0.05), while the control group showed a significant decrease in both flexibility and vertical performance^41^. The most recent study, conducted by Juric et al. investigated the effects of a HIIT intervention lasting 12 weeks on balance, coordination, speed, flexibility, strength, and agility in 10- to 15-year-old students, and showed no significant effects. This may be because short-term HIIT interventions of only two 10-min sessions per week do not provide sufficient stimulation for fitness (muscular strength, muscular endurance, power, speed, flexibility, and balance) enhancement^50^.

### Effects of different exercise intensities on MUSCLE FITNESS in children and adolescents

Five studies assessed changes in muscle fitness, and four showed that high-intensity exercise interventions had a positive impact on muscle fitness in children and adolescents. Larsen et al. explored whether the musculoskeletal fitness of 8–10 year old schoolchildren is affected by frequent high-intensity physical education classes, and showed that after a 10-month intervention of varying intensities, the intervention group had higher scores for changes in bone mineral content (BMC) and bone mineral density (aBMD) change scores were higher, suggesting that well organized high-intensity physical education sessions can promote the development of musculoskeletal fitness in young children^29^. A study of the effects of different resistance training programs on the development of muscular strength and endurance in children found a significant increase in leg extension muscular endurance with low repetition-heavy loads and high repetition-heavy loads, with high repetition-medium loads being significantly greater than low repetition-heavy loads training, and in the chest press exercise only the high repetition-medium loads exercise group had significantly greater muscular strength and muscular endurance than the control group^33^. Benson et al. found that an 8-week PRT (two sets of high-intensity exercises targeting major muscle groups) intervention resulted in significant increases in upper body strength and lower body strength compared to a control group^34^. Leppanen et al. investigated the effect of physical activity intensity on PHYSICAL FITNESS in children by using the PREFIT PHYSICAL FITNESS test to measure PHYSICAL FITNESS (that is, cardiorespiratory fitness, lower and upper body muscular strength and motor fitness), and the results showed that replacing sedentary, low- or moderate-intensity exercise with 5 min of high-intensity exercise per day promoted an increase in muscle strength^38^.

However, Videira-Silva et al. showed no significant improvement in muscular endurance in participants in the 12-week HIIT group^49^. That's because the study, which only had two 10-min short-term high-intensity interval exercise sessions per week, failed to provide enough stimulation for fitness enhancement. Therefore, long-term, high-intensity training may be necessary to effectively improve muscle fitness in children and adolescents.

## Discussion

This review aimed to summarize the effects of physical activity of different exercise intensities on the PHYSICAL FITNESS of children and adolescents. The analysis included 30 interventional studies from 15 countries. 30 studies were assessed as above average, with good reason to believe that different exercise intensities had different effects on PHYSICAL FITNESS in children and adolescents. Based on strict restrictions on the nature of the intervention included in the studies, the studies included in the study span the years 1999 to 2024 (Table 1). It can be guessed that since 1999, researchers have gradually found differences in improving the PHYSICAL FITNESS of adolescents with different exercise intensities. In addition, from the perspective of regions and countries where the literature is published, relevant research is mainly concentrated in developed countries and some developing countries. This may be because, with the increase in material wealth, the PHYSICAL FITNESS of children and adolescents has received a high level of attention. Judging from the number of relevant published literature, there is still a lack of research on the effects of different exercise intensities on the PHYSICAL FITNESS of children and adolescents internationally. Therefore, this study aims to draw the attention of more draw the attention of more researchers from different regions and countries to this topic and encourage the conduction of controlled trials with high-quality evidence to further demonstrate the positive effects of different exercise intensities.

This study shows that high intensity exercise training has significant effects in improving body composition. It was mainly more effective in reducing visceral fat. These results align with a previous review by Batacan et al., which synthesized 65 studies and showed that HIIT can significantly improve waist circumference and body fat percentage in people who are overweight or obese^54^. A meta-analysis of adolescents found that exercise interventions of different intensities were differentially effective in reducing body weight and body mass index, and that high-intensity aerobic exercise and high-intensity aerobic exercise combined with high-intensity resistance training were more effective than low- and moderate-intensity exercise interventions^55^. We suspect that this may be due to the fact that high-intensity exercise leads to excessive post-exercise oxygen consumption and the substrate for this energy oxidation is fat, during high-intensity exercise the body needs to secrete more adrenaline and noradrenaline to control the muscles, and in addition the body has to maintain high metabolic levels for a longer period of time even after exercise. All of these effects lead to an increase in the body's resting metabolic levels, which further stimulates fat burning and leads to weight loss^56,57^. It is also interesting to note that Buchan and Kargarfard, when exploring the effects of HIIT on body composition in normal and obese adolescents, did not find any good changes in body composition or waist circumference in the intervention group. Both studies claimed that the lack of effect on body composition was due to the short duration of the training (duration of 7 and 8 weeks)^58,59^. Therefore, we suggest that relevant scholars pay more attention to the optimal training time when high-intensity exercise training can effectively improve the body composition of children and adolescents, and provide more effective training programs to reduce the obesity rate of children and adolescents at home and abroad.

This study showed that both high-intensity exercise training and moderate to low-level exercise training can improve cardiorespiratory fitness in children and adolescents, but high-intensity exercise training has a more significant effect on cardiorespiratory function. This finding coincides with previous conclusions^60–62^. A meta-analysis of adolescents aged 11–17 years found that high-intensity exercise training has a significant effect on improving cardiorespiratory fitness in adolescents compared to moderate-intensity exercise^60^, which is consistent with our findings. The mechanism by which this occurs may be due to the fact that high-intensity training increases the oxidative capacity of skeletal muscle more efficiently than conventional training methods. For example, in terms of the molecular adaptive mechanisms of skeletal muscle oxidative capacity, high-intensity exercise activates the activity of AMPK and MAPK exercise-responsive kinases^63,64^, while increasing the amount of mRNA for PGC-qα, a transcription factor that regulates the oxidative function of mitochondria. With the activation of the joints leading to increased transcription of mitochondrial substances, this allows the body's aerobic and anaerobic capacity to be enhanced, leading to improved cardiorespiratory fitness^65^. We therefore recommend that schools should incorporate high-intensity program in their physical education curricula so as to improve the cardiorespiratory fitness of children and adolescents and to reduce the probability of children and adolescents suffering from cardiovascular diseases in adulthood.

Muscle fitness is widely recognized as a key fitness component for maintaining overall health and is negatively correlated with obesity^66^.In this review, five studies confirmed the effects of different exercise intensities on muscle fitness function in children and adolescents. A systematic study of school-age children and adolescents suggests that high-intensity physical activity is more beneficial in building muscle^67^. Our findings are supported by Smith et al.'s study, where strenuous physical activity was positively associated with muscle fitness in children and adolescents^68^. In addition, only 1 study in this study showed that high-intensity training was effective in improving muscle flexibility. Muscle flexibility can be expressed as the normal physiological range of joint motion^69^. If adequate flexibility is lacking, daily activities will become difficult. In addition, reduced flexibility can also lead to musculoskeletal injuries^70^. Therefore, maintaining (or increasing) flexibility is essential as it maintains normal joint motion, thereby reducing the risk of injury^71^. A study of adolescents aged 14–17 years found that a 12-week, high-intensity training intervention resulted in adolescents displaying greater flexibility^72^, which is consistent with our findings. Furthermore, in the literature included in this review, only 1 study showed that high-intensity training improves muscle flexibility, but there was insufficient evidence that muscle flexibility is associated with high-intensity training. We speculate that on the one hand, this may be related to limitations in the assessment of muscle flexibility. The currently commonly used methods of assessing muscle flexibility (sitting and stretching) are unable to detect a lack of function due to muscle laxity^73^; the other side of the coin is that most of the current research on muscle flexibility has focused on the elderly population, with less attention paid to children and adolescents. This is due to the fact that muscle flexibility decreases with age, leading to increased joint stiffness and progressive loss of balance, which increases the risk of falls in older adults^73^. Overall, appropriate levels of flexibility have positive implications for the PHYSICAL FITNESS of children and adolescents, and exploring scientifically sound methods of assessing flexibility and research on flexibility in children and adolescents should receive more attention.

### Research limitations and prospects

Although this review discusses the effects of different exercise intensities on the PHYSICAL FITNESS of children and adolescents from four aspects, its limitations should be properly examined. This review provides direction for further research on the effects of different exercise intensities on the PHYSICAL FITNESS of children and adolescents. Although an extensive literature search was conducted, including articles published before 2024, it is possible that some relevant literature may have been overlooked due to variations in keywords used in this study. Additionally, we conducted an extensive literature search in four major databases, but some published non-English foreign studies may have been missed in this review as our search was limited to English-language journal articles.

Despite these limitations, this review systematically collated the literature reports on the different effects of different exercise intensities on the PHYSICAL FITNESS of children and adolescents. Future research could explore higher-quality randomize controlled trials to provide more convincing evidence for optimal exercise intensity to improve the health of children and adolescents. Future research should also focus on the effect of different exercise intensities on muscle flexibility. At the same time, more comprehensive exercise evaluation is needed to support high-intensity exercise training as an effective exercise program to improve the PHYSICAL FITNESS of children and adolescents.

## Conclusions

This systematic review demonstrates a positive association between high-intensity exercise training and PHYSICAL FITNESS in children and adolescents. High-intensity exercise training yields notable improvement in body composition (reduced body mass index, waist circumference, and body fat), cardiopulmonary function, and muscle strength in children and adolescents. Furthermore, the high-intensity training group outperforms both the moderate-intensity group and the control group in terms of improving physical fitness. Specifically, participation in HIIT exhibits a more significant effect on improving PHYSICAL FITNESS in children and adolescents. Based on the findings, we recommend that schools optimize their physical education programs by incorporating more high-intensity physical activities, thereby promoting the healthy growth of children and adolescents through effective exercise.

Moreover, the study highlights that the effects of high-intensity physical activity on the PHYSICAL FITNESS of children and adolescents may be influenced by factors such as average age, overweight or obesity of participants. Therefore, further refinement of the study design is necessary, along with additional high-quality research, particularly randomized controlled trials, to ensure the long-term reliability of the results. Additionally, in terms of measurement of related indicators, this study primarily relies on manual measurement and automated equipment, which may introduce measurement errors. Subsequent studies could consider using more advanced instruments to assess relevant indicators of the PHYSICAL FITNESS of children and adolescents.

### Supplementary Information

Supplementary Information.

## Data Availability

Data is provided within the manuscript or supplementary information files.
