# Supplementary material for: Effects of different types of exercise intensity on improving health-related physical fitness in children and adolescents: a systematic review
Source: Sci Rep. 2024 Jun 21;14:14301. doi: 10.1038/s41598-024-64830-x (PMC11192957; doi:10.1038/s41598-024-64830-x)
Supplement: Supplementary file 1 — Supplementary Information. [file 41598_2024_64830_MOESM1_ESM.docx]

Supplementary Information

**Effects of different types of exercise intensity on improving** **health-related physical fitness in children and adolescents: A systematic review**

**Xianxian Zhou^1^, Jiayu Li ^1^, Xiaoping Jiang^1^***

^1^College of Physical Education and Health Sciences, Zhejiang Normal University, Jinhua, China, Zhejiang 321004, China

***Correspondence:**

Xiaoping Jiang

[jxp@zjnu.cn](mailto:jxp@zjnu.cn)

**1 Supplementary Tables**

**1.1 Supplementary Table S1**

**Table S1.** PRISMA checklist

| **Section and Topic** | **Item #** | **Checklist item** | **Location**  **where item is reported** |
| --- | --- | --- | --- |
| **TITLE** | | |  |
| Title | 1 | Identify the report as a systematic review. | Title page (p.1) |
| **ABSTRACT** | | |  |
| Abstract | 2 | See the PRISMA 2020 for Abstracts checklist. | p.1 |
| **INTRODUCTION** | | |  |
| Rationale | 3 | Describe the rationale for the review in the context of existing knowledge. | p.2-3 |
| Objectives | 4 | Provide an explicit statement of the objective(s) or question(s) the review addresses. | P.3 |
| **METHODS** | | |  |
| Eligibility criteria | 5 | Specify the inclusion and exclusion criteria for the review and how studies were grouped for the syntheses. | P.3-4 |
| Information sources | 6 | Specify all databases, registers, websites, organisations, reference lists and other sources searched or consulted to identify studies. Specify the date when each source was last searched or consulted. | P.3-4 |
| Search strategy | 7 | Present the full search strategies for all databases, registers and websites, including any filters and limits used. | Table S2 (Supplementary  material) |
| Selection process | 8 | Specify the methods used to decide whether a study met the inclusion criteria of the review, including how many reviewers screened each record and each report retrieved, whether they worked independently, and if applicable, details of automation tools used in the process. | P.4-5 |
| Data collection process | 9 | Specify the methods used to collect data from reports, including how many reviewers collected data from each report, whether they worked independently, any processes for obtaining or confirming data from study investigators, and if applicable, details of automation tools used in the process. | P.4-5 |
| Data items | 10a | List and define all outcomes for which data were sought. Specify whether all results that were compatible with each outcome domain in each study were sought (e.g. for all measures, time points, analyses), and if not, the methods used to decide which results to collect. | P.5 |
|  | 10b | List and define all other variables for which data were sought (e.g. participant and intervention characteristics, funding sources). Describe any assumptions made about any missing or unclear information. | P.5 |
| Study risk of bias assessment | 11 | Specify the methods used to assess risk of bias in the included studies, including details of the tool(s) used, how many reviewers assessed each study and whether they worked independently, and if applicable, details of automation tools used in the process. | P.5-6 |
| Effect measures | 12 | Specify for each outcome the effect measure(s) (e.g. risk ratio, mean difference) used in the synthesis or presentation of results. | Not applicable |
| Synthesis methods | 13a | Describe the processes used to decide which studies were eligible for each synthesis (e.g. tabulating the study intervention characteristics and comparing against the planned groups for each synthesis (item #5)). | Not applicable |
|  | 13b | Describe any methods required to prepare the data for presentation or synthesis, such as handling of missing summary statistics, or data conversions. | Not applicable |
|  | 13c | Describe any methods used to tabulate or visually display results of individual studies and syntheses. | Not applicable |
|  | 13d | Describe any methods used to synthesize results and provide a rationale for the choice(s). If meta-analysis was performed, describe the model(s), method(s) to identify the presence and extent of statistical heterogeneity, and software package(s) used. | P.5-6 |
|  | 13e | Describe any methods used to explore possible causes of heterogeneity among study results (e.g. subgroup analysis, meta-regression). | Not applicable |
|  | 13f | Describe any sensitivity analyses conducted to assess robustness of the synthesized results. | P.5-6 |
| Reporting bias assessment | 14 | Describe any methods used to assess risk of bias due to missing results in a synthesis (arising from reporting biases). | P5-6 |
| Certainty assessment | 15 | Describe any methods used to assess certainty (or confidence) in the body of evidence for an outcome. | P.5-6 |
| **Section and Topic** | **Item #** | **Checklist item** | **Location**  **where item is reported** |
| **RESULTS** | | |  |
| Study selection | 16a | Describe the results of the search and selection process, from the number of records identified in the search to the number of studies included in the review, ideally using a flow diagram. | P.5-6 and Figure 1 |
|  | 16b | Cite studies that might appear to meet the inclusion criteria, but which were excluded, and explain why they were excluded. | P.6-7 |
| Study characteristics | 17 | Cite each included study and present its characteristics. | P.7-10 and Table 1 |
| Risk of bias in studies | 18 | Present assessments of risk of bias for each included study. | P.10-11 and Table 2 |
| Results of individual studies | 19 | For all outcomes, present, for each study: (a) summary statistics for each group (where appropriate) and (b) an effect estimate and its precision (e.g. confidence/credible interval), ideally using structured tables or plots. | P.10-112and Table 2 |
| Results of syntheses | 20a | For each synthesis, briefly summarise the characteristics and risk of bias among contributing studies. | P.13-15 |
|  | 20b | Present results of all statistical syntheses conducted. If meta-analysis was done, present for each the summary estimate and its precision (e.g. confidence/credible interval) and measures of statistical heterogeneity. If comparing groups, describe the direction of the effect. | P.13-15 |
|  | 20c | Present results of all investigations of possible causes of heterogeneity among study results. | P.13-15 |
|  | 20d | Present results of all sensitivity analyses conducted to assess the robustness of the synthesized results. | P.13-15 |
| Reporting biases | 21 | Present assessments of risk of bias due to missing results (arising from reporting biases) for each synthesis assessed. | P.13-15 |
| Certainty of evidence | 22 | Present assessments of certainty (or confidence) in the body of evidence for each outcome assessed. | P.13-15 |
| **DISCUSSION** | | |  |
| Discussion | 23a | Provide a general interpretation of the results in the context of other evidence. | P.15-16 |
|  | 23b | Discuss any limitations of the evidence included in the review. | P.18 |
|  | 23c | Discuss any limitations of the review processes used. | P.18 |
|  | 23d | Discuss implications of the results for practice, policy, and future research. | P.18-20 |
| **OTHER INFORMATION** | | |  |
| Registration and protocol | 24a | Provide registration information for the review, including register name and registration number, or state that the review was not registered. | P.4 |
|  | 24b | Indicate where the review protocol can be accessed, or state that a protocol was not prepared. | P.20 |
|  | 24c | Describe and explain any amendments to information provided at registration or in the protocol. | Not applicable |
| Support | 25 | Describe sources of financial or non-financial support for the review, and the role of the funders or sponsors in the review. | P.24 |
| Competing interests | 26 | Declare any competing interests of review authors. | P.24 |
| Availability of data, code and other materials | 27 | Report which of the following are publicly available and where they can be found: template data collection forms; data extracted from included studies; data used for all analyses; analytic code; any other materials used in the review. | Supplementary material: data extracted from include studies; and data used for all analyses. |

**1.2 Supplementary Table S2**

**Table S2. Search strategies**

| Web of science =6926 | AB=(Adolescent OR child* OR schoolchildren OR preschooler OR preadolescent OR teen* OR youth) AND AB=(exercise OR physical*activity OR physical*education OR fitness OR sport* OR motor ) AND AB=( strength OR flexibility OR motor OR endurance OR agility OR body composition OR anthropometry OR body mass index OR waist circumference OR overall adiposity OR central adiposity OR overweight OR obesity OR risk factors OR risk score cardiovascular disease OR metabolic syndrome OR blood glucose OR glucose tolerance OR insulin resistance OR insulin sensitivity OR blood lipids OR dyslipidemia OR diabetes OR blood pressure OR hypertension OR inflammatory markers OR bone mineral OR bone mineral content) AND AB=(random OR random controlled OR controlled trial OR trial) |
| --- | --- |
| Pub-Med =175 | (Adolescent[Title] OR child*[Title] OR schoolchildren[Title] OR preschooler[Title] OR preadolescent[Title] OR teen*[Title] OR youth[Title]) AND (exercise[Title] OR physical*activity[Title] OR physical*education[Title] OR fitness[Title] OR sport*[Title] OR motor[Title])) AND (strength[Title] OR flexibility[Title] OR motor[Title] OR endurance[Title] OR agility[Title] OR body composition[Title] OR anthropometry[Title] OR body mass index[Title] OR waist circumference[Title] OR overall adiposity[Title] OR central adiposity[Title] OR overweight[Title] OR obesity[Title] OR risk factors[Title] OR risk score cardiovascular disease[Title] OR metabolic syndrome[Title] OR blood glucose[Title] OR glucose tolerance[Title] OR insulin resistance[Title] OR insulin sensitivity[Title] OR blood lipids[Title] OR dyslipidemia[Title] OR diabetes[Title] OR blood pressure[Title] OR hypertension[Title] OR inflammatory markers[Title] OR bone mineral[Title] OR bone mineral content[Title])) AND (random[Title] OR random controlled[Title] OR controlled trial[Title] OR trial[Title]) |
| Scopus =2023 | (TITLE-ABS-KEY ( Adolescent OR child* OR schoolchildren OR preschooler OR preadolescent OR teen* OR youth ) AND TITLE-ABS-KEY ( exercise OR "physical activity" OR "physical education" OR fitness OR sport* OR motor ) AND TITLE-ABS-KEY ( strength OR flexibility OR motor OR endurance OR agility OR "body composition" OR anthropometry OR "body mass index" OR "waist circumference" OR "overall adiposity" OR "central adiposity" OR overweight OR obesity OR "cardiovascular disease" OR "metabolic syndrome" OR "blood glucose" OR "glucose tolerance" OR insulin OR "blood lipids" OR dyslipidemia OR diabetes OR "blood pressure" OR hypertension OR "inflammatory markers" OR "bone mineral" OR "bone mineral content" ) AND TITLE-ABS-KEY ( random OR random controlled OR "controlled trial" OR trial ) ) |
| EBSCO=902 | TI (adolescent OR child* OR schoolchildren OR preschooler OR preadolescent OR teen* OR youth) AND TI (exercise OR physical*activity OR physical*education OR fitness OR sport* OR motor) AND TI (strength OR flexibility OR motor OR endurance OR agility OR body composition OR anthropometry OR body mass index OR waist circumference OR overall adiposity OR central adiposity OR overweight OR obesity OR risk factors OR risk score cardiovascular disease OR metabolic syndrome OR blood glucose OR glucose tolerance OR insulin resistance OR insulin sensitivity OR blood lipids OR dyslipidemia OR diabetes OR blood pressure OR hypertension OR inflammatory markers OR bone mineral OR bone mineral content) AND TI (random OR random OR controlled OR controlled OR trial OR trial) |

**1.3 Supplementary Table S3**

**Table S3.Testing tools**

| **Author / Year** | **Tool** |
| --- | --- |
| Farah (2014) | Fili Zola scale；Wall mounted stadiometer；Fili Zola; Peak oxygen uptake (VO2peak); commercial enzyme-linked immunosorbent multiplex panel assay kits; Heart-rate monitor |
| Hay (2016) | Intravenous glucose tolerance test; Magnetic resonance spectroscopy; Dual X-ray absorptiometry-derived; polar heart rate monitor; Dina map automatic machine |
| Bond (2015) | Flow mediated dilation (FMD); laser Doppler imaging (peak reactive hyperemia; PRH); ECG-gating software |
| Paravidino (2016) | Heart rate monitors; portable electronic scale; portable stadiometer; triaxial accelerometers |
| Tadiotto (2023) | portable stadiometer; Colorimetric enzymatic method; K4b2 metabolic analyzer |
| Larsen (2018) | Whole-body dual-energy X-ray absorptiometry (DXA); Flamingo balance and standing long jump;20-m sprint tests |
| Ramirez-Velez (2022) | Cytokine antibody array; Heart rate monitors |
| Cao (2022) | Dual-energy x-ray absorptiometry; anthropometry; DXA; Blood sample collection; TANITA scale; automatic BP monitor; non-deformable tape; Fasting blood draws; automated device |
| Dias (2018) | Maximal exercise test; magnetic resonance imaging (MRI); dual-energy X-ray absorptiometry (DXA); air-displacement plethysmography |
| Faigenbaum (1999) | Child-size dynamic constant external resistance (DCER) equipment |
| Benson (2008） | Portable stadiometer; digital scale and BIA used standardized techniques; height-based treadmill walk protocol; automated computerized breath-by-breath metabolic cart |
| Taber (2014) | Accelerometers、calibrated stadiometer、dual-energy X-ray absorptiometry、BMI |
| Davis (2012) | Oral glucose tolerance test; via dual-energy X-ray absorptiometry; magnetic resonance; glucose oxidase method |
| Burns (2012) | Electronic wall-mounted stadiometer; scale; flexible measuring tape; calipers; automated metabolic cart; automated sphygmomanometer |
| Leppanen (2016) | WGT3x-BT triaxial accelerometer; air-displacement plethysmography |
| Leppanen (2017) | wGT3x-BT triaxial accelerometer; air-displacement plethysmography |
| Gomes (2013) | Use an open-circuit boli code element analyzer |
| Buchan (2013) | Calibrated electronic weighing scales; automated monitor; opt jump system; electronic sprint timer with photoelectric sensors; continuous heart rate telemetry; enzymatic methods |
| Grasten (2022) | 20mSRT test; Actigraphy GT3X+ waist-worn activity monitors |
| Costigan (2015) | Progressive Aerobic Cardiovascular Endurance Run shuttle test; portable digital scale; portable stadiometer; non-extensible steel tape |
| Saidi (2019) | Anthropometric; body composition; Yo-Yo intermittent recovery level 1 tests; Happened Skinfold Caliper; Horne–Oseberg morningness-eveningness questionnaire (MEQ) the self-report Pittsburgh sleep quality index (PSQI); Epworth sleepiness scale (ESS) questionnaires; heart rate monitor; Met Amax 3B portable gas analyzer; standard ergometer software |
| Saidi (2020) | Pittsburgh Sleep Quality Index; Pediatric Daytime Sleepiness Scale; Heart rate monitor; dual-energy X-ray absorptiometry; electromagnetically braked cycle ergometer |
| Gerber (2021) | Light triaxial accelerometers; SPSS version 26; ACTi life software |
| Winn (2021) | Asthma Control Questionnaire; Electromagnetically braked cycle ergometer |
| Paulino (2021) | Forearm plethysmography; Rating perceived exertion; Yo-Yo Intermittent Endurance Test level one; electrical weight scale; portable stadiometer; self-reported questionnaire – the International Physical Activity Questionnaire |
| Videira-Silva (2022) | Dual-energy X-ray absorptiometry; Appropriately sized cuff; Gas analyzer |
| Juric (2023) | Anthropometer; Tanita BC- 418 Segmental Body Composition Analyzer |
| Farpour-Lambert (2009) | Electronic scale; Hardened stadiometer; Dual-energy X-ray absorptiometry; Validated automated device |
| Ketelhut, Sascha  et al. (2024) | Heart rate monitor;Dual-energy X-ray absorptiometry;Body mass index z-score (BMI-z) |
| [JairoH. Migueles,PhD](https://jamanetwork.com/searchresults?author=Jairo+H.+Migueles&q=Jairo+H.+Migueles)(2023) | automatic sphygmomanometer (M6; Omron)；via dual-energy x-ray absorptiometry (Discovery Horizon DXA system; Hologic Inc)；Accelerometers (GT3X+; ActiGraph) |
